# Society Reports—Medico-Chirurgical Society

**Published:** 1878-08-20

**Authors:** 


### SOCIETY REPORTS.

### MEDICO-CHIRURGICAL SOCIETY.

ATLANTA, July, 1878.

Dr. Crawford in the chair.

Dr. R. C. Word introduced the subject of Cholera-Infantum, and asked the views of the members in regard to the nature and treatment of this affection. He regarded the so-called summer complaint of children, as the same disease, differing only in degree, or as modified by the difference of location and state of the atmosphere. He asked for information especially as to the treatment of the disease. He did not care to have recommended the old routine method of calomel, opium and astringents. In some cases the disease recovered under this treatment, and in exceptional cases it might recover under almost any treatment. But as a rule he regarded this method as a failure. The percentage of mortality in this disease had been, and is yet, fearful, especially in our large cities. He had, in a great degree, abandoned the old method. Occasionally he prescribed a mercurial at the start, but relies mostly upon the use of the bromides, oft repeated; aconite in minute and repeated doses, where there is fever; sometimes gelseminum, tepid or cold bath, cold cloths or soothing applications to the abdomen; cold water to drink, *ad libitum*, and as ant-acid and restraining, the improved Chalk Mixture, as published in the June number of THE SOUTHERN MEDICAL RECORD, or the Neutralizing Cordial, in the same number of journal. Under this treatment his success in summer complaints of children had been much better than formerly, yet not altogether satisfactory.

For children deprived of the breast, he had found condensed milk to be the best article of diet. The "Eagle brand," he thought the best, though not always uniform as to the quantity of sugar it contained, being sometimes too sweet.

He did not propose to discuss the subject fully, but desired to elicit the views of the members.

Dr. A. R. Alley said that cholera infantum was generally endemic to the Gulf and South Atlantic and sea coast cities, where it prevails sometimes almost epidemically with fearful mort lity. It may occur away from these points in a limited and sporadic form, being modified by the cooler atmosphere.

As to its etiology, the profession seems to be in doubt as to whether it is produced by heat and atmospheric condition or by dentition. The profession generally, he thought, accepted the theory that it was due to heat aggravated by dentition. The symptoms of cholera infantum are the same as Asiatic cholera in the adult—vomiting and purging, often ending in collapse and death.

Away from the larger central points we have the summer complaints of children from teething, etc. He regarded heat and atmospheric condition as the prime causes of cholera, dentition being only a secondary cause, or perhaps only aggravating the disease by keeping up constantly the irritation. His plan of treating the disease, when not in the aggravated form, is to do very little, relying upon hygienic rules and good nourishing food with stimulants. If the bowels are very loose he had used the following with fine success:

| | | |
|---|---|---|
| R.—Subnitrate bismuth.......... | 1 to 2 | grains. |
| Pepsin...................... | 1 to 2 | " |
| Tannin...................... | ½ to 2½ | " |
| Pulv. cinnamon............ | ½ to 2 | " |

As a dose to be given every two hours. Using also hot stupes of mint and flannel over the whole abdominal region. He never gives calomel in this disease at the early stage, as advised by some, as it tends to increase the irritation and nausea. There being a tendency to collapse it acts as a sedative, and no doubt produces congestion of the brain.

He never gave mercury until the stools denote the absence of bile, and then only in small stimulating doses of ¼ to ⅓ grain. He "expunged" the chalk mixture and hydrarg cum. creta as they cause irritation and increase the vomiting. His treatment was rather *expectant*. He never gave cold water, but pellets of ice, which is more grateful and does not overload the stomach. If the child be too weak to nurse, he used the condensed milk, Eagle brand, as follows: a large tablespoonful is added to a tumblerful of hot water and the solution fed to the child by a spoon. If the skin be hot and dry use warm bath, adding whisky to it. Let the child be lightly clothed and kept cool, with free ventilation, along with Valentine meat juice which is a splendid preparation, being light, nutrititious and ready for use when needed.

If the gums are red and swollen, they should be lanced, and if painful rub them with a mixture of laudanum and honey in small quantities, applied with the finger. It will be found very soothing to the gums.

Dr. T. S. Powell said that cholera infantum was, in all probability, caused by impure air. It seemed to be a species of cholera peculiar to this country, and found mostly in the larger cities of the Middle and Southern States, occurring only in the summer season, by which we infer that a high temperature is among the causes which develop the disease. Teething children are most subject to it, and many have attributed the disease to this cause alone.

True cholera infantum presents in the outset, all the symptoms of cholera morbus in the adult, but the prognosis is less favorable, and the disease, if not speedily terminating in collapse, is protracted, attended with diarrhœa and a remittent form of fever, with intense thirst and a tendency to cerebral congestion, convulsions, etc.

Post mortems reveal congestion but seldom inflammation of the brain. Occasionally there is inflammation and hydrocephalus, but inflammation of the mucous membrane of the stomach and bowels is seldom, if ever, absent; and the liver is almost invariably engorged, sometimes to an enormous extent.

We have been struck by the resemblance of the liver as it appears in chickens that die of chicken cholera to the enlarged liver of a child which has died of cholera infantum.

In regard to treatment, we have seen nothing in all the new agents proposed of late years, which improves greatly upon that proposed by Dewers and certain of the older writers. It is true that cholera infantum is an exceedingly fatal disease, and the percentage of deaths is great, and must be so, especially in view of the fact that the most important remedial measure suggested by the physician is seldom carried out because of poverty or other inability of the parents, and that is to remove the child from the impure, heated atmosphere of the city to the purer and cooler air of the country.

As to drugs, we think that small doses of calomel, from $\frac{1}{4}$ to $\frac{1}{8}$ of a grain, is indicated in the outset. It may be well, in some cases, to encourage the vomiting at first, by draughts of tepid water to make sure that all irritating substances have been removed from the stomach; and sometimes the nausea may be allayed by injections of warm water into the rectum and repeated until a faecal or bilious discharge is obtained. When this occurs the nausea is usually suspended, and if it recur the injection may be repeated often with similar good results. Then the small doses of calomel may appropriately follow, giving a dose every two hours until the liver is acted upon, as indicated by bilious discharges, after which the calomel should be given at longer intervals. If there be not too much fever an opiate may be injected into the rectum at night to procure rest and quiet.

This treatment may be persevered in for a reasonable time, or until the secretions be established. If the disease persists the warm bath, soothing applications and such remedies as the particular symptoms of the case may indicate should be used. But in any stage of the case, if it be possible, remove the child to the country.

Dr. J. J. Knott said: I regard this affection of infants as being dependent on the depressing effects of vitiated air, and a high temperature during that peculiar period, dentition. This depression takes place in the great sympathetic, producing a condition of the alimentary canal unfavorable to assimilation. Within the last few years I have managed all the cases that I have had with but little trouble. My main reliance is in the following prescription:

R.—Lemon juice........................ oz. ii.

Carb. ammonia.................. grs. xl.

M. S. $\frac{1}{3}$ to $\frac{1}{2}$ teaspoonful every 2 or 3 hours. If the depression is very great, I give immediately 5 grs. sulph. quinine in solution by enema, rub the spine with turpentine, with turpentine applications over the bowels. If the child is nursing take it from the breast and feed it on teas until the unfavorable symptoms disappear.

---

### THE ETIOLOGY OF MEMBRANOUS DYSMENORRHŒA.

At a meeting of the Obstetrical Society of London, Dr. Cory recorded a case which strongly supported Dr. Hausmann's view that such are due to imperfect impregnation. The patient, previous to her marriage, at the age of thirty, had never passed any membrane. She aborted three times, between the second and third months, during the first two years of married life. Since then, she had almost invariably passed, at her menstrual periods, membranes, which proved to be very perfect casts of the uterine cavity, and presenting all the naked-eye and microscopical appearances of its mucous lining. The membrane usually came away on the second day of menstruation, previous to which the dysmenorrhœa was acute. Later on, she lived apart from her husband for nine months, during which time she had menstruated regularly without passing any membrane.—*Medical and Surgical Reporter.*

